# A Round Trip to the Desert: *In situ* Nanopore Sequencing Informs Targeted Bioprospecting

**DOI:** 10.3389/fmicb.2021.768240

**Published:** 2021-12-13

**Authors:** Adriel Latorre-Pérez, Helena Gimeno-Valero, Kristie Tanner, Javier Pascual, Cristina Vilanova, Manuel Porcar

**Affiliations:** ^1^Darwin Bioprospecting Excellence S.L., Paterna, Spain; ^2^Institute for Integrative Systems Biology I2SysBio (University of València-CSIC), Paterna, Spain

**Keywords:** Nanopore sequencing, bioprospecting, *in situ* sequencing, 16S rRNA gene sequencing, microbiome analysis, Tabernas Desert

## Abstract

Bioprospecting expeditions are often performed in remote locations, in order to access previously unexplored samples. Nevertheless, the actual potential of those samples is only assessed once scientists are back in the laboratory, where a time-consuming screening must take place. This work evaluates the suitability of using Nanopore sequencing during a journey to the Tabernas Desert (Spain) for forecasting the potential of specific samples in terms of bacterial diversity and prevalence of radiation- and desiccation-resistant taxa, which were the target of the bioprospecting activities. Samples collected during the first day were analyzed through 16S rRNA gene sequencing using a mobile laboratory. Results enabled the identification of locations showing the greatest and the least potential, and a second, informed sampling was performed focusing on those sites. After finishing the expedition, a culture collection of 166 strains belonging to 50 different genera was established. Overall, Nanopore and culturing data correlated well, since samples holding a greater potential at the microbiome level also yielded a more interesting set of microbial isolates, whereas samples showing less biodiversity resulted in a reduced (and redundant) set of culturable bacteria. Thus, we anticipate that portable sequencers hold potential as key, easy-to-use tools for *in situ*-informed bioprospecting strategies.

## Introduction

Scaling laws have predicted that the Earth is home to 1 trillion (10^12^) microbial species (Locey and Lennon, 2016). A large fraction of this biodiversity still remains to be explored and very likely harbors novel molecules, enzymes and/or biological activities with potential applications in industrial processes, drug development, cosmetics or environment-related issues (i.e., bioremediation). The search for these novel products from biological sources and, in particular, from microorganisms, is known as microbial bioprospecting. Extreme environments, such as the deep sea or hyper-arid deserts, are of special interest for bioprospecting studies, as they tend to be sources of undiscovered biodiversity (Bull and Goodfellow, 2019).

The characteristics (i.e., nutrient and oxygen availability, humidity, irradiation, pH, etc.) of a given environment shape the composition of its microbiota, often leading to the existence of temporal and spatial variations in the microbial community composition (Lauber et al., 2009; DiGiulio et al., 2015). Spatial changes have also been observed at microscale: for example, in gradients of soil depths as recently demonstrated with the SoilBox system (Bhattacharjee et al., 2020).

In this context, sequencing technologies can be used for elucidating whole microbial profiles from samples, thus enabling to unveil changes in microbiome composition which are usually not detected with culture-based approaches. Illumina sequencing platforms—such as the MiSeq System—are the current standard for microbiome sequencing. Nevertheless, this technology is time-consuming and usually requires shipping the samples to a centralized sequencing facility. Therefore, *in situ* third-generation sequencing (TGS) strategies emerge as a promising alternative to this traditional approach (Latorre-Pérez et al., 2021).

Among TGS technologies, the Oxford Nanopore Technologies (ONT) MinION system is especially relevant for *in situ* sequencing as it is the smallest sequencing device currently available, it is inexpensive in comparison to other TGS devices, and the obtention of long reads can be assessed in real-time (Latorre-Pérez et al., 2021). Thus, sequencing data can be directly analyzed through bioinformatic pipelines that can be run on servers, laptops, or even mobile phones (Mitsuhashi et al., 2017; Palatnick et al., 2021).

Nanopore sequencing has previously been used in range of real-time applications, such as pathogen detection and surveillance (Quick et al., 2016; Charalampous et al., 2019; Chan et al., 2020); forensic identification (Tytgat et al., 2020; Vasiljevic et al., 2021); or industrial process monitoring (Hardegen et al., 2018; McHugh et al., 2021). Among all the potential uses of MinION, *in situ* sequencing is especially interesting for those situations where no alternative analyses are feasible due to a lack of equipment (i.e., second-generation sequencing platforms, qPCR instruments.). This is the case for most bioprospecting expeditions, which are usually carried out far away from microbiology laboratories. Previous works have demonstrated that both sample preparation and microbiome sequencing can be achieved using a reduced, mobile laboratory. Indeed, Nanopore sequencing has been successfully applied in extremely remote locations such as the Antarctic Dry Valleys (Johnson et al., 2017), the Canadian High Arctic (Goordial et al., 2017), the largest European ice cap (Vatnajökull, Iceland) (Gowers et al., 2019) or the International Space Station (Castro-Wallace et al., 2017; Burton et al., 2020). Beyond the undoubtedly scientific interest of analyzing microbial samples up to hundreds of kilometers away from the nearest laboratory, microbial bioprospecting could further benefit from *in situ* sequencing, as it would allow for a more directed and evidence-based sampling procedure focused on those sampling locations that prove to be enriched with the microbial taxa and/or biological activities of interest.

To test this hypothesis, we planned a two-night expedition to the Tabernas Desert (Almería, Spain). This dryland has recently been reported to harbor a previously unexplored high bacterial biodiversity, significantly enriched in radiation- and desiccation-resistant microorganisms, which were the target of our study (Molina-Menor et al., 2021). A minimum setup of both laboratory and bioinformatic tools was designed and used for analyzing biocrust and soil samples *via* 16S rRNA gene sequencing throughout the expedition. The obtained taxonomic profiles were used to identify sample types enriched in taxa that have been described to be radiation resistant, allowing us to collect additional samples before ending the journey. Overall, this work demonstrates the feasibility of using portable, nanopore-based sequencing devices to study microbial communities without the need of returning to the lab, which could potentially inform decision-making during sampling.

## Results

### Sampling Expedition Roadmap

**FIGURE 1:**
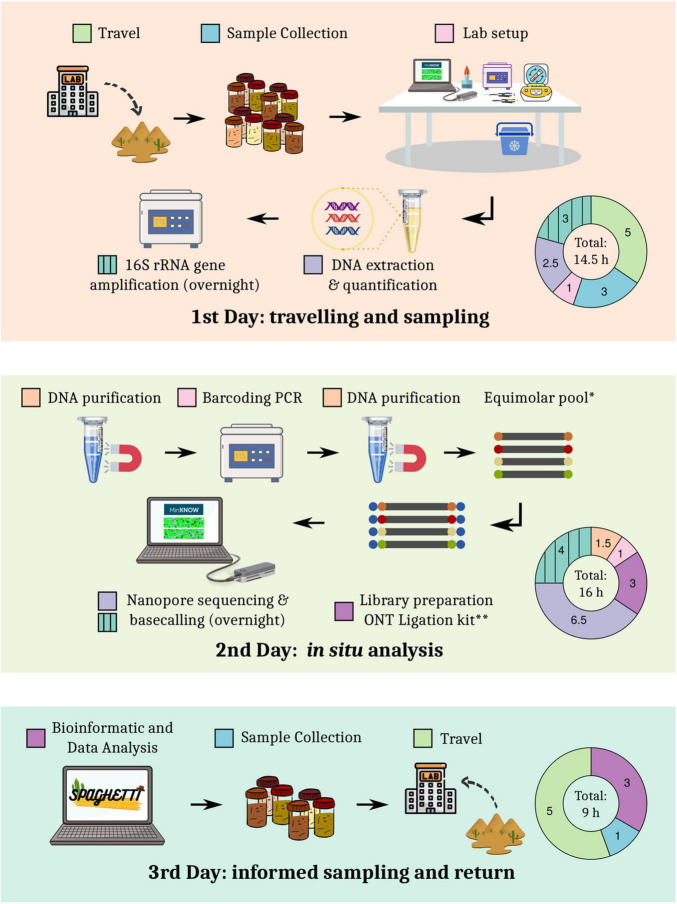
Detailed roadmap of the sampling expedition.

Based on previous sampling experiences in the Tabernas Desert and sequencing tests performed in the laboratory, a detailed roadmap for the expedition and the experimental procedures was designed (Figure 1). The total duration of the expedition was less than 60 h, including traveling (∼25% of hands-on time) and two nights. The rest of hands-on time was spent on library preparation (∼28%), sequencing and basecalling (∼26%), sampling and setup (13%), and data analysis (8%). The first set of sequencing data was generated approximately 24 h after sample collection.

### Microbiome Sequencing and Bioinformatic Analysis

Twelve biocrust and two bulk soil samples (“control” samples) were collected and analyzed through full-length 16S rRNA gene sequencing using the ONT MinION platform. A total of 1,657,804 raw reads were generated. After length and quality filtering, an average of 101,972 ± 20,949 sequences per sample were obtained (min: 50,051; max: 128,282; median *Q* = 10.3). Reads were subsequently analyzed by using a custom pipeline (Spaghetti), which was inspired by previous works (Cuscó et al., 2018; Santos et al., 2020; Urban et al., 2021). Spaghetti relied on minimap2 (Li, 2018) alignments against the SILVA v. 138 database (Quast et al., 2013), and taxonomic assignments were obtained in ∼2 h. Other alignment tools were tested as alternatives to minimap2, but they were discarded for different reasons: BLAST took ∼26 h to finish a ∼1M reads analysis, while LAST exceeded the available laptop’s RAM (16 Gb).

### Taxonomic and Diversity Analysis

Spaghetti data analysis and visualization pipeline generated several plots designed to provide a rapid overview of the taxonomy and the diversity of the samples (Supplementary File 1). At the phylum level, biocrust samples were dominated by *Cyanobacteria* (∼34.5% of average relative abundance), *Bacteroidota* (∼22.7%), *Proteobacteria* (∼19.2%), *Acidobacteriota* (∼6.0%) and *Actinobacteriota* (∼4.7%), while soil samples were mainly characterized by *Actinobacteriota* (∼24.8%), *Acidobacteriota* (∼18.6%), *Proteobacteria* (∼14.2%). *Planctomycetota* (∼14.2%) and *Gemmatimonadota* (∼7.3%) (Supplementary Figure 1 and Supplementary Table 1).

**FIGURE 2:**
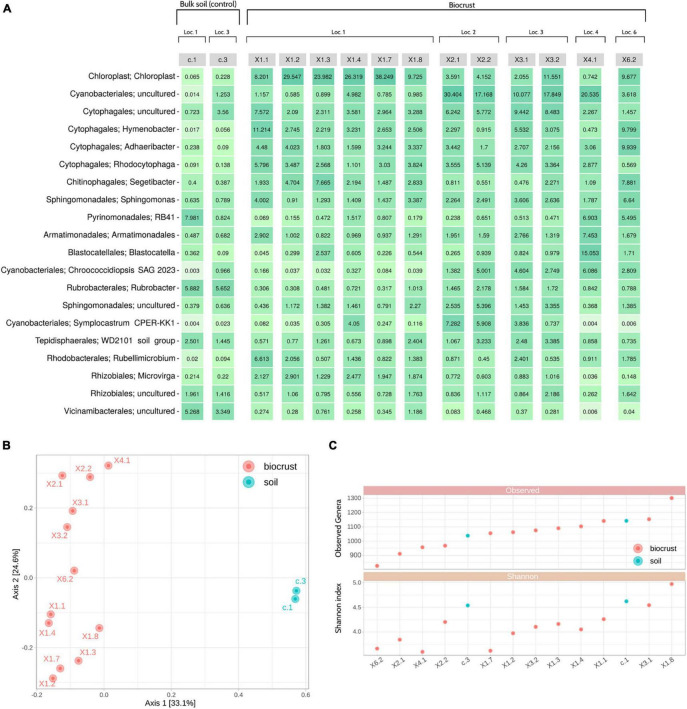
Nanopore sequencing results. **(A)** Heatmap showing the top 20 genera detected in the samples and their relative abundances. **(B)** Principal Coordinates Analysis (PCoA) using the Bray-Curtis dissimilarity metric. **(C)** Alpha diversity analysis: Observed genera (richness) (top); Shannon index (bottom). Samples are ordered by richness. Loc, Location.

As expected, a higher variability in the microbiome composition was detected at the genus level, with an uncultured *Cyanobacteriales* (∼4.7% of average relative abundance), *Hymenobacter* (∼3.9%), an uncultured *Chroococcidiopsaceae* (∼3.8%), an uncultured *Spirosomaceae* (∼3.7%) and *Adhaeribacter* (∼3.5%) being the most dominant taxa for biocrust samples. Moreover, a considerable amount of reads (∼14.0%) were assigned to chloroplasts in these samples. On the other hand, soil samples were mainly characterized by *Rubrobacter* (∼5.8%), *Vicinamibacteraceae* (∼4.8%), an uncultured *Pirellulaceae* (∼4.7%), *Pyrinomonadaceae* RB41 (∼4.4%), an uncultured *Vicinamibacterales* (∼4.1%), and a low presence of reads assigned to chloroplasts (∼0.15%) (Figure 2A and Supplementary Table 2).

Beta diversity analyses showed that biocrust and soil samples were clearly distinguishable at the microbiome level. Moreover, samples tend to cluster based on their sampling location (X1, X2, X3, X4 or X6), instead of other characteristics (i.e., color and shape of the biocrust) (Figure 2B). Alpha diversity indices were used to identify the most and least rich and diverse samples, which were X1.8/X1.3/c.1 and X6.2/X2.1/X4.1, respectively (Figure 2C).

### Radiation- and Desiccation-Resistant Bacteria Detection

Once the general taxonomic and diversity profiles were obtained, special attention was paid to 29 bacterial genera that had proven to be radiation- and/or desiccation-resistant according to the literature (Montero-Calasanz et al., 2013; Yu et al., 2015; Deng et al., 2016; Etemadifar et al., 2016; Paulino-Lima et al., 2016; Golinska et al., 2020; Tanner, 2020). The objective of this analysis was to identify those samples which maximized the richness and abundance of those radiation- and/or desiccation- resistant taxa, since they should hold a greater potential for isolating and discovering microbial strains and substances of biotechnological interest.

**FIGURE 3:**
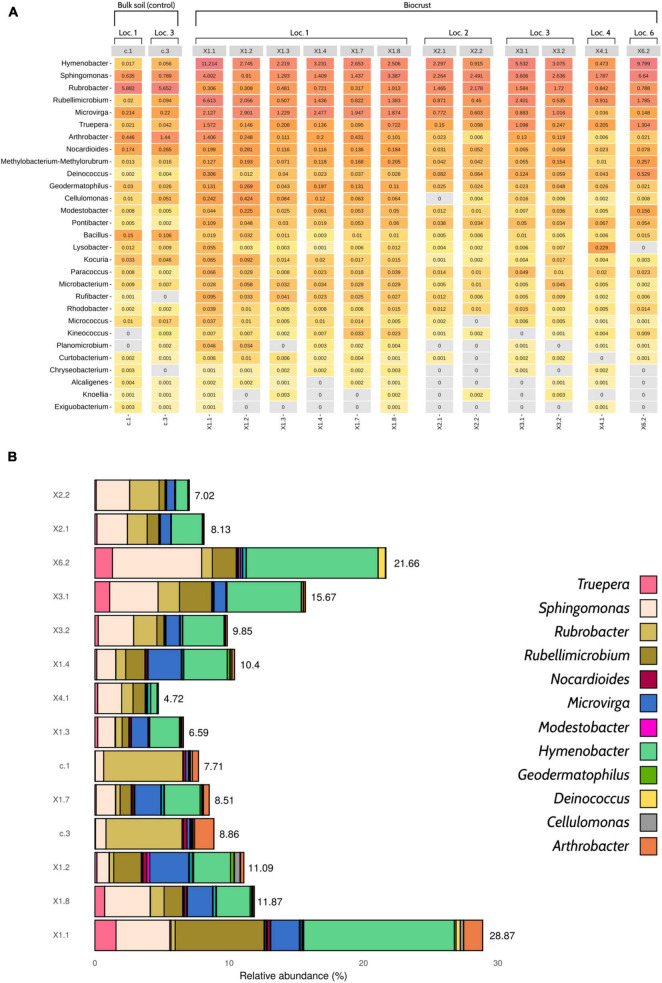
Profile of desiccation- and radiation-resistant bacteria according to Nanopore sequencing data. **(A)** Heatmap showing the 29 genera of interest and their relative abundances (%). **(B)** Barplot displaying the cumulative relative abundances of the selected taxa (*n* = 29). Only 12 genera have been colored in order to improve visualization, as the abundance of some taxa was so low that they cannot be properly distinguished in the figure. An interactive version of this figure including the 29 genera of interest can be found in Supplementary Figure 4. The relative abundance of desiccation- and radiation-resistant genera was calculated considering the whole microbial community, not only the taxa of interest. Loc, Location.

Overall, the number of radiation- and desiccation-resistant genera detected in the samples by Nanopore sequencing was high, ranging from 23 (X2.1 and X2.2) to 29 (X1.1 and X1.8) (Figure 3A). Although some of the taxa were present in low abundance (< 0.01%), the selected bacteria accounted for 11.5% of the relative abundance of the samples, in average (Figure 3B). Biocrust profiles were dominated by *Hymenobacter* (∼3.9% of the total relative abundance), *Sphingomonas* (∼2.7%), *Rubellimicrobium* (∼1.7%), *Microvirga* (∼1.3%) and *Rubrobacter* (∼1%). The two bulk soil samples were mainly characterized by the presence of *Rubrobacter* (∼5.8%), *Arhtrobacter* (∼0.9%) and *Sphingomonas* (∼0.7%) (Figure 3 and Supplementary Table 2).

After analyzing all the results provided by the pipeline, additional samples were collected. This time, bioprospecting activities focused on obtaining biological replicates of three selected samples: (a) biocrust X1.1, with the highest number of radiation- and desiccation-resistant genera (29); (b) bicrust X2.1, with the lowest number of radiation- and desiccation-resistant genera (23); and (c) bulk soil c.1, taken as a control for comparisons between biocrust and bulk soil samples.

GPS positions of the original samples were traced back and samples were identified based on the pictures that were taken on the first sampling day. Finally, two additional replicates were collected for each type of sample.

### Microbial Collection Establishment and Identification

Back in the laboratory after the expedition, all the collected samples (*n* = 20) were cultured under three different conditions: (1) Tryptic Soy Agar (TSA) medium, (2) SSE/HD medium (SSE/HD), and (3) SSE/HD medium + uninterrupted artificial light (SSE/HD + light). A total of 166 strains comprising 50 different genera were isolated and identified through Sanger sequencing of the partial 16S rRNA gene. The bacterial colonies displayed differences in morphology and appearance, with white, yellow, pink, red, orange and brown being the most predominant colors (Supplementary Table 3). Initially, samples cultured on SSE/HD + light did not display any microbial growth after 4 weeks of incubation. For that reason, plates were removed from the artificial light, and a few days later, different bacterial colonies started to grow.

**FIGURE 4:**
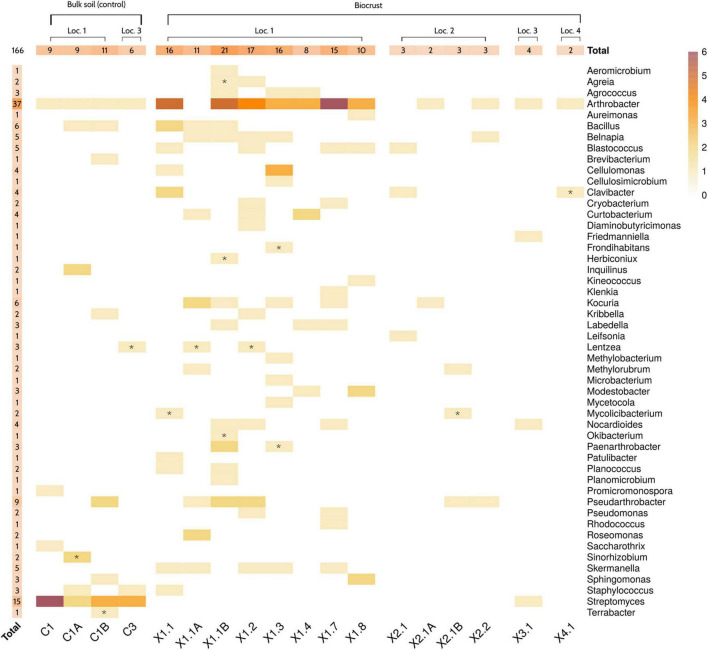
Culture collection description. Heatmap showing the number of strains isolated from each sample. Genus-level taxonomy of the strains was obtained by partial 16S rRNA gene sequencing of isolates. Letters “A” and “B” indicate the samples that were collected on the third day, after analyzing the original samples by Nanopore sequencing. Symbol “*” highlights those genera that were not originally detected in that sample by *in situ* sequencing. Only genera with a relative abundance higher than 0.001% were considered as detected. Samples 3.2 and 6.2 are not shown, since no bacterial strain was isolated from them. Loc, Location.

The genus *Arthrobacter* was the most represented in the microbial collection, with up to 37 isolates belonging to this taxonomic group (Figure 4). A total of 15 strains, which were mainly isolated from soil samples, were classified as *Streptomyces.* Other predominant genera in the collection were *Pseudoarthrobacter* (9 isolates), *Kocuria* (6), *Bacillus* (6), *Skermanella* (5), *Blastococcus* (5), and *Belnapia* (5). At the sample level, biocrusts collected from Location 1 (X1) presented the highest number of bacterial isolates. Specifically, samples X1.1B (21 isolates/15 unique genera), X1.2 (17/13), X1.3 (16/12), and X1.1 (16/10) showed the highest diversity of cultured bacteria. On the other hand, samples X3.2 (0/0), X6.2 (0/0), X4.1 (2/2), and X2.1A (2/2) presented the lowest diversity of isolates (Figure 4).

The taxonomic profiles obtained by Nanopore sequencing were compared to the results from the molecular identification of the isolated strains. Overall, Nanopore sequencing and culture-based data correlated well. In fact, only 14 out of the 166 isolated strains belonged to genera that were not detected in the original sample by *in situ* microbiome sequencing (Figure 4 and Supplementary Table 3). Interestingly, three of the isolated genera (*Mycolicibacterium, Lentzea*, and *Sinorhizobium*) were not detected in any sample of the dataset. After revising the database used for assigning the taxonomy of the reads (see section “Materials and Methods”), a mislabeling of those taxa at the genus level was detected. Specifically, *Mycolicibacterium* was labeled as *Mycobacterium, Lentzea as Lechevalieria* and *Sinorhizobium* as *Ensifer*. These three genera were indeed detected by Nanopore sequencing in all the samples where the strains were isolated from Supplementary Table 2. Finally, it is worth highlighting that some of the most abundant radiation-resistant bacteria detected by *in situ* 16S rRNA sequencing (i.e., *Hymenobacter, Rubrobacter, Rubellimicrobium, Microvirga, Truepera*…) were not cultured from any sample. Indeed, only 50 out of the 441 genera (11.3%) with an average relative abundance > 0.01% according to Nanopore sequencing were represented in the microbial culture collection.

**FIGURE 5:**
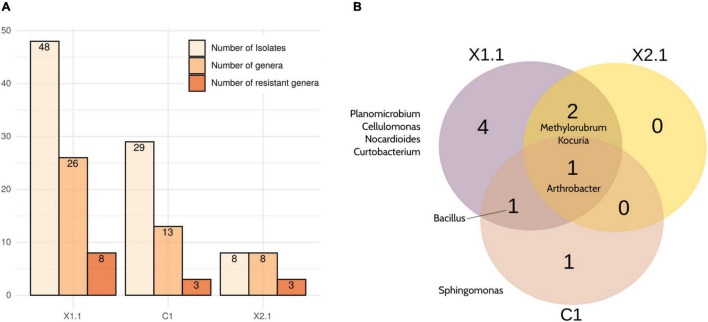
Summary of the strains isolated from the selected samples (X1.1, X2.1, and C1). **(A)** Number of different strains, genera and desiccation-/ radiation- resistant genera isolated from each sample. **(B)** Venn diagram comparing the desiccation- and radiation-resistant genera isolated from any replicate of each selected sample. Note that all the identifications were obtained by partial 16S rRNA gene Sanger sequencing.

Among the selected samples, X1.1 yielded the highest number of total cultured strains (48), the highest number of different cultured genera -as deduced by partial 16S rRNA gene Sanger sequencing- (26), and the highest number of cultured genera classified as radiation- or desiccation-resistant according to literature (8) (Figure 5). In contrast, and as expected considering the results from *in situ* sequencing (Figure 3B), X2.1 samples displayed the lowest diversity of cultured bacteria and radiation- and desiccation-resistant genera. Moreover, almost all the genera isolated from X2.1 samples were also isolated from X1.1 (Figure 5B, Supplementary Figure 2, and Supplementary Tables 4, 5), thus confirming the hypothesis that this sample was less valuable from the bioprospecting point of view. A different profile of bacteria was isolated from C1 bulk soil samples (Supplementary Figure 2), with only one radiation-resistant genus -*Sphingomonas-* cultured exclusively from this type of sample (Figure 5B). Interestingly, the relative abundance of *Sphingomonas* was higher in all the biocrust samples than in bulk soil (Figure 3A), although this genus was isolated only from samples C1B and X1.8.

**FIGURE 6:**
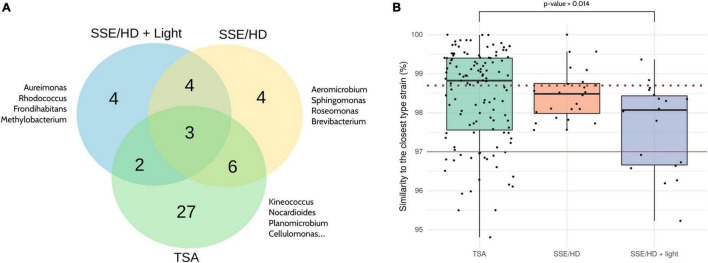
Comparison of the different culture conditions. **(A)** Venn diagram showing the bacterial genera isolated from each culture condition. The complete list of genera isolated from each condition can be found in Supplementary Table 6. **(B)** Percentage of similarity shared by each strain and its closest phylogenetic neighbor according to partial 16S rRNA gene sequencing. The dotted and the solid red lines are drawn on 98.7 and 97% of similarity, respectively. The Mann–Whitney *U* test was applied for comparing between groups, and *p*-values were corrected using the Benjamini-Hochberg method. Only significant results are highlighted. Note that all the identifications were obtained by partial 16S rRNA gene Sanger sequencing.

Focusing on the culture conditions, 124 strains were isolated from TSA (38 different genera), 24 from SSE/HD (17 different genera), and 18 from SSE/HD + light (13 different genera) (Figure 6 and Supplementary Table 6). Nevertheless, strains isolated from SSE/HD + light presented a significantly lower similarity to their closest type strain than strains isolated from TSA (FDR adjusted *p*-value < 0.05; Mann–Whitney *U* test), based on partial 16S rRNA gene sequencing. In fact, ∼89% of the strains isolated from SSE/HD + light showed a similarity lower than 98.7% to their closest neighbor, a common threshold for defining new species (Chun et al., 2018), compared to ∼46 and 66% displayed by TSA- and SSE/HD-isolated strains, respectively (Figure 6B).

## Discussion

Bioprospecting is often a unidirectional process, with scientists leaving their research institute for several days or weeks to collect samples that are only screened upon arrival at the laboratory. This is usually a blind task, since the screening results are obtained once the expedition is over. As sampling sites are generally remote and far from the researcher’s laboratory, returning to the locations where bioprospecting occurred is not always viable, thus preventing further exploitation of the samples that showed a greater potential based on the screening. This work is a proof of concept of the use of portable Nanopore sequencing as a tool for guiding and informing bioprospecting activities during a sampling expedition, in our case to the only European desert, the Tabernas Desert (Almería, Spain).

ONT sequencing is a well-established technique for studying microbial communities (Ciuffreda et al., 2021), and portable sequencing (i.e., MinION) has indeed been applied to characterize microbiomes in some of the most remote places of the universe that are accessible to human beings (Castro-Wallace et al., 2017; Goordial et al., 2017; Johnson et al., 2017; Gowers et al., 2019; Burton et al., 2020). Although some authors have demonstrated the utility of *in situ* sequencing to assess the animal biodiversity in the rainforest (Menegon et al., 2017; Pomerantz et al., 2018), the present work is, to the best of our knowledge, the first confirmation that this technology can be applied during a microbial bioprospecting expedition to improve the bioprospecting strategy itself.

Our results demonstrate that DNA analyses can be integrated into the sampling roadmap, while keeping the duration of the journey under 72 h (Figure 1). The obtained sequencing yield was substantially higher than the output reported in other on-site studies (Latorre-Pérez et al., 2021), and it was comparable to the yield of runs performed on fully equipped laboratories (Nygaard et al., 2020; Urban et al., 2021). It must be noted that instead of directly sequencing in the field, we decided to set up a mobile laboratory 15 km away from the sampling location in an apartment with internet and electricity access. This allowed us to apply the same protocols that we routinely use in the laboratory with little modifications, thus reducing the risk of failure during the expedition. Nevertheless, simplified protocols (i.e., Field Sequencing Kit; ONT, Oxford, United Kingdom, Cat. No.: SQK-LRK001) involving shorter preparation time and less equipment could be employed, even with the lack of electricity or internet, as has been previously demonstrated (Edwards et al., 2019; Gowers et al., 2019). Indeed, Spaghetti does not require an internet connection, so this pipeline could be also used for on-site analyses.

Different sample types (i.e., biocrust and bulk soil) were clearly distinguishable according to microbial profiles (Figure 2). As expected, *Cyanobacteria* was more abundant in biocrust samples, since these microorganisms are a crucial part of biological soil crusts, which often also harbor other organisms such as lichens, microalgae, microfungi or mosses (Williams et al., 2016; Machado-de-Lima et al., 2019). This would explain the higher presence of sequences assigned to chloroplasts in this type of samples. Overall, phylum-level taxonomy was concordant with the microbial profiles expected for soil samples, with *Bacteroidota, Proteobacteria, Acidobacteriota, Actinobacteriota, Planctomycetota, Verrucomicrobiota*, and *Gemmatimonadota* dominating the microbiomes (Buckley et al., 2006; Spain et al., 2009; Bergmann et al., 2011; DeBruyn et al., 2011; Zhang et al., 2019; Kalam et al., 2020; Larsbrink and McKee, 2020). At the genus level, differences and similarities between samples were resolved. In consequence, *in situ* Nanopore sequencing could be especially helpful for choosing those samples that maximize the microbial diversity -according to beta diversity or any other metric-, preventing the selection of samples with poor diversity or little variation for further screening, thus saving time and resources.

Taxonomic information could also be used for identifying those samples that contain the microorganisms of interest. As a proof of concept, we focused on genera that were previously described to be desiccation- and/or radiation-resistant, and which thus hold potential for biotechnological applications (Gabani and Singh, 2013; Molina-Menor et al., 2021). The prevalence of these taxa in the samples collected from the Tabernas Desert was high (Figure 3). This was expected, since most of these bacteria are often found in or isolated from other arid soils and biocrusts (Holmes et al., 2000; Rainey et al., 2005; Zhang et al., 2007; Abed et al., 2010; Amin et al., 2016; Hu et al., 2016; Wübbeler et al., 2017; Liang et al., 2019). Nevertheless, *in situ* sequencing in combination with our analysis pipeline led to the categorization and identification of samples that showed a greater diversity and abundance of the genera of interest. Thanks to such information, those samples -technically, biological replicates of the samples- could be further collected and thoroughly analyzed back in the laboratory.

It is well known that detecting a certain taxon by high-throughput sequencing does not necessarily mean that this taxon can be successfully isolated from the sample. In our case, culture-based and Nanopore sequencing data correlated well (Figure 4), although an important fraction of the genera detected with the sequencing approach was not represented in the microbial culture collection. This could be expected given that a significant number of prokaryotic taxa are virtually “unculturable.” In any case, the sample that held the greatest potential at the microbiome level -according to Nanopore data- (X1.1) also resulted in the most interestingly complex set of culturable bacteria (Figure 5), despite using a relatively simple culturing approach. On the other hand, some of the most dominant bacteria according to sequencing data could not be isolated from any sample (i.e., *Hymenobacter* or *Rubrobacter*) very likely due to culturing biases. Although this limitation is inherent to bioprospecting strategies that rely on obtaining microbial cultures, knowing the presence of a certain taxonomic group in the sample would allow for the use of microorganism-specific culture conditions or enrichment methods, thus increasing the chances of success.

In general, the profile of bacteria isolated from the Tabernas Desert was similar to the one previously described (Molina-Menor et al., 2021). *Arthrobacter* was the predominant genus, which is consistent with the observations of da Rocha et al. (2015). Other bacteria, such as *Belnapia, Kocuria* or *Skermanella* were also recurrent in biocrust samples. Nevertheless, up to 29 genera isolated in this study were not recovered by Molina-Menor et al. (2021).

Interestingly, some of the isolated bacteria may represent new species according to partial 16S rRNA gene sequencing, showing the great, yet to be discovered, ecological and biotechnological potential hidden in the Tabernas Desert. Although full 16S rRNA gene sequences and genomes should be retrieved for circumscribing new taxa (Chun et al., 2018), bacteria isolated from SSE/HD + light displayed a lower similarity to any other previously described type strain (Figure 6). These results were indeed obtained by serendipity, as bacterial growth was only detected after removing the culture plates from artificial light (∼4 weeks after plating), which was not the original idea.

Despite the promising results obtained in this proof of concept, we have identified some limitations of *in situ* Nanopore sequencing. The first one is the taxonomic resolution of 16S rRNA gene sequencing. Although long-read platforms have the ability to sequence the full-length 16S rRNA gene, the intrinsic error associated to ONT sequencing hampers species-level identification. This error also hinders the direct comparison between Nanopore-based microbiome sequencing and the 16S rRNA gene sequences obtained from the isolates by Sanger sequencing, as it would be difficult to discern if a particular fraction of Nanopore reads actually comes from a specific strain in the collection or from a phylogenetically related strain (or even species) that may or may not have been isolated. For that reason, we decided to perform the analyses at the genus level and to compare the taxonomic profiles instead of comparing the sequences. Nanopore-based, 16S rRNA gene sequencing has proved to be robust for microbiome characterization at this taxonomic level, showing a performance similar to Illumina sequencing (Cuscó et al., 2018; Heikema et al., 2020; Matsuo et al., 2020; Nygaard et al., 2020; Winand et al., 2020). However, as the final objective of bioprospecting is to actually isolate the bacterial strains, it must be noted that phenotype can greatly vary among members of the same genus or even species, so genus-resolved taxonomy could be insufficient in some cases. Recent studies have shown that species-level resolution is feasible thanks to advances in software (Curry et al., 2021; Rodríguez-Pérez et al., 2021), while other works demonstrated that improved taxonomic resolution could be achieved by using longer amplicons (16S-ITS-23S) (Benítez-Páez and Sanz, 2017; Cuscó et al., 2018). Moreover, Nanopore sequencing errors are also decreasing due to improvements in basecallers and chemistries, which have allowed to reach up to 99.3% of modal accuracy on raw reads (accessed on July 17, 2021).^1^ If accuracy continues to increase at this rate, it is reasonable to think that species-level identifications, and even strain-level resolution in some cases, may be achieved in the near future. Nevertheless, high-accuracy basecalling models are based on complex machine learning methods that require longer execution time, so improvements on the speed of these models are still required for being used in real-time applications (Xu et al., 2020).

It must be highlighted that this study was focused on the detection and isolation of potential radiation- and desiccation-resistant bacteria according to their taxonomic affiliation and according to the previous bibliography describing this type of features in particular genera. Our approach is thus a proof of concept that a wide taxonomic group can be identified in the samples by using Nanopore sequencing, but 16S rRNA gene itself would not be an accurate predictor of the actual ability of the isolates to resist radiation or desiccation (Steen et al., 2019). If the purpose of the bioprospecting expedition is to detect specific functional activities, shotgun metagenomic data would be needed to resolve the taxonomy at the strain level (Dilthey et al., 2019) and to ascertain the functional potential of the different members of the microbial community according to their gene content. In this regard, it has to be noted that ONT sequencers tend to incorporate indel errors on the reads that complicate the functional prediction (Watson and Warr, 2019; Latorre-Pérez et al., 2020), and this is therefore a current limitation of the informed bioprospecting strategy we are describing in this work.

Finally, sequencing strategies show the microbiome composition based on relative abundances, which may mislead the results interpretation. For instance, if a taxon is detected in Sample 1 and in Sample 2 at 10 and 1% of relative abundance, respectively, that does not imply that Sample 1 has a higher absolute abundance of the target bacteria, since the total microbial load of the samples has not been measured. This should be taken into account when selecting the samples of interest for further exploitation.

Notwithstanding the limitations, our results clearly show that Nanopore sequencing is a powerful tool for deciphering the microbial composition of different samples during a bioprospecting expedition, and that it can contribute to optimize the sampling strategy *in situ*. With microorganisms colonizing almost any known biotope (Archer et al., 2019; Sielaff et al., 2019; Tanner et al., 2020), an instrument able to resolve microbial communities inhabiting different niches is a valuable resource that can be used for targeting sample collection. Therefore, it can be envisaged a close future in microbial ecology, in which bioprospecting journeys will start with a preliminary sampling step, coupled to nanopore-based *in situ* analysis, which will enable a second, more targeted sampling (of specific plant species, soil depths, geological substrates, salt concentration, humidity level, etc.) in a very short time lapse. This strategy will both ease further work in the lab and increase the chances of identification of the target microbial taxa and/or biomolecule of interest.

## Materials and Methods

### Sample Collection

Sampling was carried out in November 2020 at the Tabernas Desert Natural Park (Almeria, Spain), under the permission of the competent authorities. Biocrust and bulk soil samples were collected in two different days. Biocrust samples were gathered using a laboratory spatula that was sterilized with ethanol 96% immediately before collecting each sample. Bulk soil (∼5 cm deep) was directly introduced into sterile falcon tubes. On the first day, fourteen different samples were taken, and then analyzed through *in situ* microbiome sequencing. Based on the results, six additional samples were gathered in the second sampling day. These samples were, indeed, biological replicates of the least and most promising samples based on sequencing data. Metadata (geolocation, type of sample, appearance and pictures) was collected and associated to each sample (Supplementary Figure 3).

### Laboratory Setup

Requirements for DNA extraction, PCR amplification, library preparation and sequencing were evaluated, and a minimum laboratory setup was designed accordingly (Supplementary Table 7). The necessary equipment fitted in the trunk of a compact car, and it was transferred to an apartment in Viator (Almería, Spain), 15 km away from the Tabernas Desert, where the mobile laboratory was established and all the experimental and data analysis procedures were carried out. The apartment was equipped with electricity, internet connection, a fridge and a freezer.

### DNA Extraction and 16S rRNA Gene Amplification

Approximately 0.25 g of the samples were used to perform DNA extraction with the DNEasy Power Soil Kit (QIAGEN, Germany, Cat. No.: 12888) according to the manufacturer’s instructions, with an additional incubation step at 65°C after the addition of the C1 solution. DNA was resuspended in 30 μL of sterile Mili-Q water. Qubit x1 dsDNA High-Sensitivity Assay kit (Qubit 2.0 Fluorometer, Thermo Fisher, Waltham, United States, Cat. No.: Q33230) was used for DNA quantification. PCR amplification of the full-length bacterial 16S rRNA gene (V1-V9; ∼1.45 kbp) was carried out by using the S-D-Bact-0008-a-S-16 (5′-AGR GTT YGA TYM TGG CTC AG-3′) and S-D-Bact-1492-a-A-16 (5′-TAC CTT GTT AYG ACT T-3′) primers (Klindworth et al., 2013), which were tailed with the ONT Universal Tags: 5′-TTT CTG TTG GTG CTG ATA TTG C-3′ for forward primer, and 5′-ACT TGC CTG TCG CTC TAT CTT C-3′ for reverse primer. The PCR reaction mix for each sample consisted of 22 μL of H_2_O, 25 μL of NZYTaq II 2x Green Master Mix (NZYTech, Lisboa, Portugal, Cat. No.: MB358), 1 μL of both forward and reverse primers and 1 μL of template DNA. For the negative control, 1 μL of Mili-Q water was used instead. The following conditions were used for PCR: initial denaturation (94°C; 1 min); amplification (35 cycles) comprising denaturation (95 °C; 1 min), annealing (49 °C; 1 min) and extension (72°C; 2 min); final extension (72 °C; 10 min). The resulting amplicons were purified with the NucleoMag kit for PCR clean up with magnetic beads (Macherey-Nagel, Germany, Cat. No.: 744100.4). Magnetic beads were used at 0.5 x concentration, and manufacturer’s instructions were followed.

Barcodes were added by employing the PCR Barcoding Expansion Pack 1-96 (ONT, Oxford, United Kingdom, Cat. No.: EXP-PBC096). PCR mix consisted of 22 μL of H_2_O, 25 μL of NZYTaq II 2x Green Master Mix, 1 μL of the specific barcode and 2 μL of the purified DNA. The following conditions were used for PCR: initial denaturation (95°C; 3 min); amplification (15 cycles) comprising denaturation (95°C; 15 s), annealing (62°C; 15 s) and extension (72°C; 90 s); final extension (72°C; 5 min). Amplicons were purified with the NucleoMag kit and quantified with the Qubit x1 dsDNA High-Sensitivity Assay kit. Finally, an equimolar pool of amplicons was prepared for library construction.

### Library Preparation and Nanopore Sequencing

The Ligation Sequencing Kit (ONT, Oxford, United Kingdom, Cat. No.: SQK-LSK109) was used to prepare the sequencing library. Briefly, the NEBNext FFPE DNA Repair Mix (New England Biolabs, Ipswich, United States, Cat. No.: M6630) was used for DNA repair and end-prep. Then, a purification with the NucleoMag kit was carried out. Finally, adapter ligation and clean-up was performed by following the ONT SQK-LSK109 protocol.

A R9.4.1 MinION flow cell (ONT, Oxford, United Kingdom, Cat. No.: FLO-MIN106D) was primed and loaded as indicated by the manufacturer. Sequencing was performed during ∼6.5 h. Reads were basecalled with MinKNOW software (v. 20.06.5; core v. 4.0.5) using Guppy’s (v. 4.0.9) fast basecalling model, and sequences with *Q* < 7 (default threshold implemented in MinKNOW) were discarded.

### Bioinformatic and Statistical Analysis

Reads were analyzed with Spaghetti, a custom pipeline for automatic bioinformatic analysis of Nanopore sequencing data and semi-automatic exploratory analysis and data visualization. Briefly, Spaghetti bioinformatic pipeline consists of the following steps:

1. Porechop (v. 0.2.4)^2^ is run with default parameters for removing sequencing adapters from reads.
2. Nanofilt (v. 2.7.1) (De Coster et al., 2018) is used to filter reads shorter than 1,200 bp or longer than 1,800 bp.
3. Quality check is carried out with NanoStat (v. 1.4.0) using default parameters (De Coster et al., 2018).
4. Chimeras are detected and removed by using yacrd (v. 0.6.2) with -c and -n parameters set to 4 and 0.4, respectively, as suggested by the authors for Nanopore data (Marijon et al., 2020).
5. Filtered reads are mapped against the SILVA database (v. 138) (Quast et al., 2013), as formatted and provided by Qiime2,^3^ by using minimap2 (v. 2.17-r9419) (Li, 2018) with “-x map-ont” and “–secondary = no” options. In order to reduce minimap2’s memory usage, -K option was set to 10M, as previously suggested (Gamaarachchi et al., 2019).
6. Alignments are subsequently filtered with in-house python scripts (included in the pipeline), and taxonomy and abundance tables are obtained.

A detailed explanation of the pipeline and the specific commands that were used can be found on Spaghetti’s GitHub repository.^4^

Spaghetti data visualization and analysis module was mainly based on the phyloseq R package (v. 1.30.0) (McMurdie and Holmes, 2013). For alpha diversity tests, all the samples were rarefied to the lowest library size (50,051 reads/sample) to mitigate uneven sequencing depth. For beta diversity, Principal Coordinates Analysis (PCoA) were created using the Bray-Curtis dissimilarity metric and relative abundances. Heatmaps were produced with ampvis2 (v. 2.6.5) (Andersen et al., 2018). Custom figures were created using ggplot2 (v. 3.3.1). Plotly (v. 4.9.2.1) was used for producing interactive plots. Venn diagrams were obtained using an online tool.^5^

All the analyses were run on a MSI GF63 Thin 9SC-047XES laptop (CPU: Intel Corei7-9750H, 6 core, 12 threads; RAM: 16GB; SSD: 512 Gb; Graphics Card: GeForce GTX 1650).

### Isolation of Bacterial Strains

Upon arrival at the laboratory, the samples were homogenized by mixing 1 g of the sample with 1 mL of sterile Phosphate Buffered Saline (PBS) and serial dilutions up to 10^–7^ were performed. Then, 50 μL of the 10^–2^ to 10^–7^ dilutions were spread on Petri dishes containing either TSA medium (composition in g/L: 15.0 tryptone, 5.0 soya peptone, 5.0 sodium chloride, 15.0 agar) or SSE/HD 1:10 medium (composition detailed on the DSMZ media database, medium number 1,426). In the case of the SSE/HD 1:10 medium, duplicates of each dilution were cultured, with one of the replicates being incubated under uninterrupted artificial light and the other replicate being incubated, together with the TSA plates, under natural light. All plates were incubated in oxygenic conditions and at room temperature.

Individual colonies were selected based on their color and morphology from the TSA and SSE/HD 1:10 plates incubated under natural light after 6, 11, 18, 30 and 35 days of incubation (Supplementary Table 3). These colonies were re-streaked on fresh culture medium to isolate them in pure culture. Most of the isolates were obtained from TSA medium and from the more concentrated dilutions (10^–2^ and 10^–4^). Regarding the samples cultured on SSE/HD 1:10 under uninterrupted artificial light, these were removed from the artificial light after 4 weeks of incubation as they did not display any microbial growth. A few days after removal, different bacterial colonies started to grow. These colonies were re-streaked on fresh culture medium and isolated in pure culture. All pure strains were cryo-preserved in glycerol (20% glycerol in an over-night culture of the strain) at −80°C for further uses.

### Molecular Identification of Isolates

A loopful of each isolate, grown on solid medium, was resuspended in 100 μL of sterile Milli-Q water and subjected to a rapid DNA extraction that consisted of three cycles of boiling and freeze-thawing. Then, a PCR was performed to amplify the 16S rRNA gene using the following universal primers: 8F (5′-AGAGTTTGATCCTGGCTCAG-3′) (Edwards et al., 1989) and 1492R (5′-GGTTACCTTGTTACGACTT-3′) (Stackebrandt and Liesack, 1993). The following conditions were used for PCR: initial denaturation (95°C; 5 min); amplification (24 cycles) comprising denaturation (94°C; 15 s), annealing (48°C; 15 s) and extension (72°C; 90 s); final extension (72°C; 5 min).

Amplicons were visualized by electrophoresis in a 1% agarose gel stained with GoldView DNA Safe Stain (UVAT Nerium Scientific, Valencia, Spain) (100 V, 30 min). Amplicons were precipitated overnight at –20°C in a mixture of isopropanol 1:1 (vol:vol) and potassium acetate 1:10 (vol:vol) (3M, pH 5). The next day, DNA was pelleted by centrifugations for 10 min at 12,000 rpm, then washed with 70% ethanol and resuspended in 15 μL of sterile Milli-Q water. Amplicons were tagged using the BigDye^®^ Terminator v3.1 Cycle Sequencing Kit (Applied Biosystems, Carlsbad, CA, United States) and sent for Sanger sequencing of the partial 16S rRNA gene at the SCSIE (Serveis Centrals de Suport a la Investigació Experimental) of the University of Valencia (Spain), using the same universal primers as previously mentioned (8F and 1492R).

All resulting sequences were edited with UGENE v.33 (Okonechnikov et al., 2012) to remove low quality base calls, and taxonomic identification was performed using the BLASTn tool and the 16S ribosomal RNA sequences (Bacteria and Archaea) database (NCBI). Finally, clones were dereplicated using the BLASTn tool to compare each partial 16S rRNA sequence to the rest of strains belonging to the collection of microorganisms established in this project. Any strain displaying > 99.9% similarity to another strain in the collection and isolated from the same sample was considered to be a replicate and therefore discarded from the collection. This was performed to avoid an overestimation of the culturable diversity, as bacterial clones of the same species are not relevant for the microbial collection. The comparison between results from Nanopore sequencing and microbial culture collection was based on taxonomic information. Nanopore and Sanger 16S rRNA gene sequences were taxonomically classified independently, as described above. Then, the genus-level profiles were evaluated to find those taxa that had been identified by both approaches.

## Data Availability Statement

Nanopore raw sequences have been deposited in the NCBI (BioProject ID: PRJNA749463). Spaghetti is available on GitHub (https://github.com/adlape95/Spaghetti).

## Author Contributions

AL-P and HG-V performed the in-field experimental and bioinformatic work. AL-P and JP performed the data analysis, while HG-V and KT established, and characterized the microbial culture collection. AL-P prepared the figures. AL-P, MP, and CV designed the experiment and the expedition. All the authors wrote and revised the manuscript.

## Conflict of Interest

The authors declare that the research was conducted in the absence of any commercial or financial relationships that could be construed as a potential conflict of interest.

## Publisher’s Note

All claims expressed in this article are solely those of the authors and do not necessarily represent those of their affiliated organizations, or those of the publisher, the editors and the reviewers. Any product that may be evaluated in this article, or claim that may be made by its manufacturer, is not guaranteed or endorsed by the publisher.

## Footnotes

1
https://nanoporetech.com/accuracy

2
https://github.com/rrwick/Porechop

3
https://docs.qiime2.org/2020.8/data-resources/

4
https://github.com/adlape95/Spaghetti

5
http://bioinformatics.psb.ugent.be/webtools/Venn/
