# Supplementary material for: A Round Trip to the Desert: In situ Nanopore Sequencing Informs Targeted Bioprospecting
Source: Front Microbiol. 2021 Dec 13;12:768240. doi: 10.3389/fmicb.2021.768240 (PMC8710813; doi:10.3389/fmicb.2021.768240)
Supplement: Supplementary file 1 [file Data_Sheet_1.zip › Supplementary Figure S1.PDF]

	Bulk soil (control)		Biocrust											
	Loc. 1	Loc. 3	Loc. 1						Loc. 2		Loc. 3		Loc. 4	Loc. 6
	c.1	c.3	X1.1	X1.2	X1.3	X1.4	X1.7	X1.8	X2.1	X2.2	X3.1	X3.2	X4.1	X6.2
Cyanobacteria	0.151	3.857	14.339	34.277	30.67	53.306	42.205	13.356	58.025	45.779	32.633	39.106	32.019	17.903
Bacteroidota	5.355	5.311	38.803	22.36	27.289	14.508	17.64	24.838	17.813	16.207	26.386	21.318	12.274	32.673
Proteobacteria	15.44	13.041	27.181	22.523	14.045	15.29	21.215	26.641	12.855	16.485	17.424	19.304	8.65	28.516
Acidobacteriota	25.82	11.35	1.114	1.771	6.984	3.91	4.081	6.865	1.134	3.51	3.004	3.06	29.293	7.867
Actinobacteriota	18.865	30.78	5.471	6.795	3.778	5.244	5.232	7.555	2.992	3.958	3.818	4.647	2.819	3.731
Planctomycetota	12.648	15.703	1.572	1.345	2.784	1.352	2.164	5.644	1.369	4.328	3.744	4.683	1.337	0.905
Armatimonadota	1.17	1.384	3.139	1.295	1.205	1.203	1.192	2.006	2.208	1.904	3.148	1.586	7.792	1.896
Verrucomicrobiota	4.37	2.565	0.733	2.066	5.047	0.918	1.276	1.746	0.552	3.876	1.782	1.74	0.087	0.082
Gemmatimonadota	6.799	7.759	0.618	0.674	0.615	0.673	0.603	1.831	0.665	0.827	0.617	0.98	1.067	1.4
Myxococcota	2.317	1.289	2.105	3.943	2.082	1.671	2.026	2.012	0.377	0.613	2.233	0.495	0.268	0.052
Chloroflexi	2.521	1.924	0.553	0.431	0.636	0.645	0.516	1.231	0.83	0.679	1.117	1.014	2.465	0.522
Abditibacteriota	0.474	0.277	1.085	0.778	2.37	0.344	0.568	0.924	0.184	0.368	1.011	0.445	1.169	2.253
Patescibacteria	1.056	2.151	0.171	0.306	0.712	0.26	0.406	1.64	0.23	0.517	0.628	0.442	0.091	0.042
Deinococcota	0.032	0.05	1.891	0.158	0.251	0.162	0.136	0.77	0.235	0.168	1.243	0.323	0.262	1.85
Bdellovibrionota	0.321	0.271	0.659	0.324	0.721	0.168	0.276	0.625	0.187	0.234	0.536	0.476	0.048	0.023
Firmicutes	0.428	0.952	0.236	0.537	0.105	0.091	0.137	0.179	0.11	0.112	0.132	0.133	0.113	0.118
Fibrobacterota	0.053	0.041	0.019	0.092	0.152	0.029	0.061	1.664	0.059	0.13	0.193	0.009	0.004	0.001
Nitrospirota	0.657	0.707	0.023	0.014	0.024	0.014	0.009	0.034	0.011	0.02	0.027	0.011	0.011	0.008
Desulfobacterota	0.137	0.115	0.075	0.129	0.069	0.054	0.075	0.103	0.024	0.038	0.097	0.039	0.041	0.022
SAR324_clade(Marine_group_B)	0.057	0.089	0.023	0.031	0.126	0.016	0.019	0.064	0.02	0.07	0.05	0.05	0.025	0.023

Supplementary Figure 1. Heatmap showing the top 20 phyla detected in the samples and their relative abundances. Loc. = Location.
