# Supplementary material for: A Round Trip to the Desert: In situ Nanopore Sequencing Informs Targeted Bioprospecting
Source: Front Microbiol. 2021 Dec 13;12:768240. doi: 10.3389/fmicb.2021.768240 (PMC8710813; doi:10.3389/fmicb.2021.768240)
Supplement: Supplementary file 1 [file Data_Sheet_1.zip › Supplementary Figure S2.PDF]

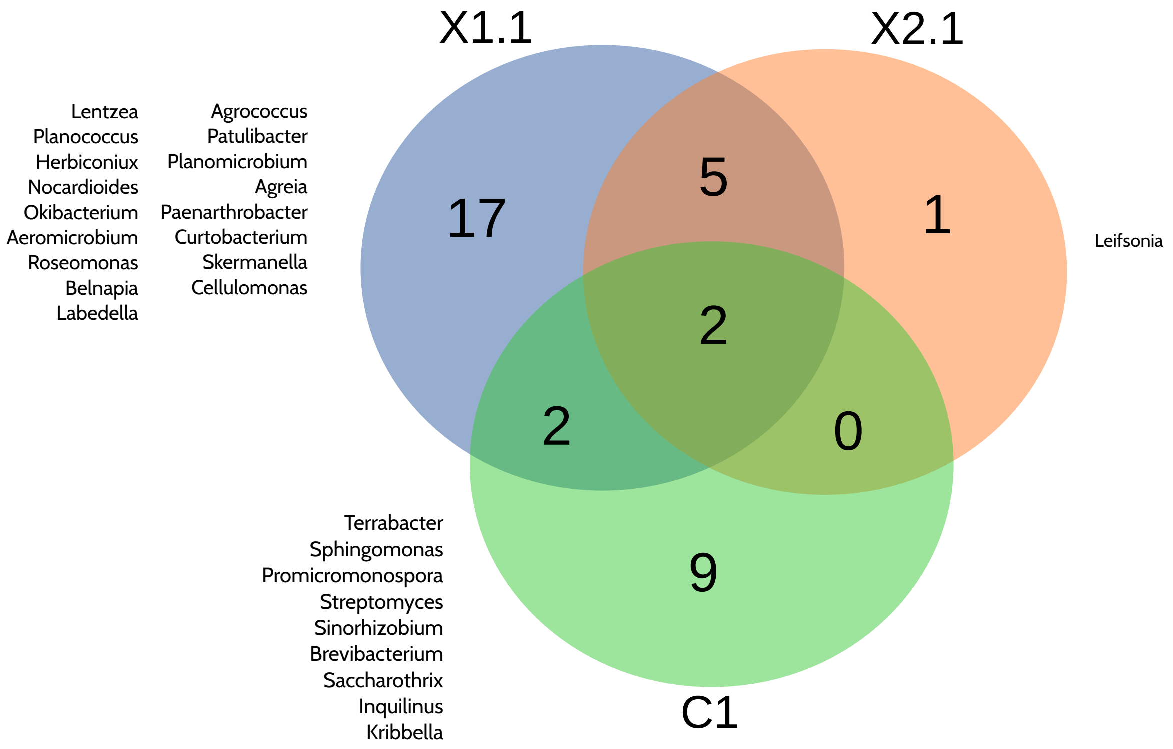

Supplementary Figure 2. Venn diagram showing the genera isolated from any replicate of each sample.
