# Supplementary material for: A Round Trip to the Desert: In situ Nanopore Sequencing Informs Targeted Bioprospecting
Source: Front Microbiol. 2021 Dec 13;12:768240. doi: 10.3389/fmicb.2021.768240 (PMC8710813; doi:10.3389/fmicb.2021.768240)
Supplement: Supplementary file 1 [file Data_Sheet_1.zip › Supplementary Figure S3.PDF]

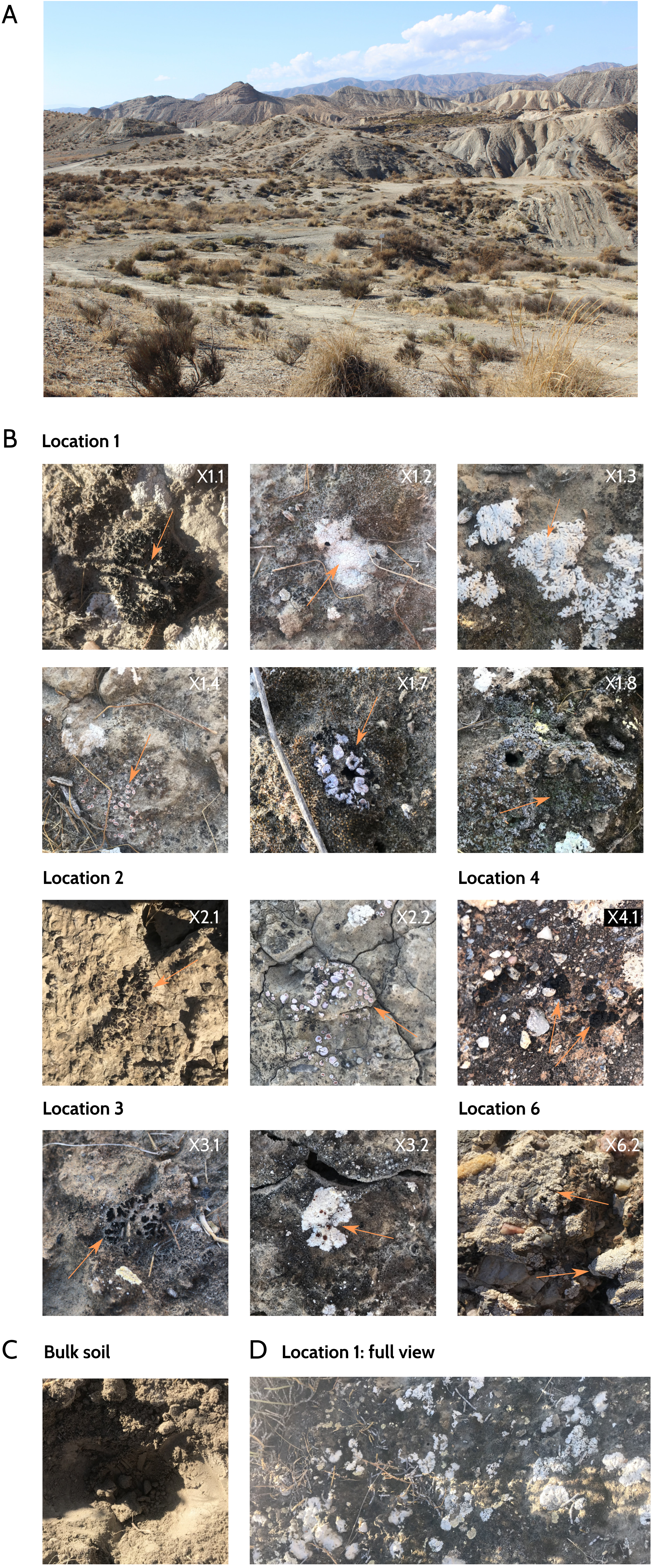

Supplementary Figure 3. Sampling overview. (A) Tabernas Desert landscape. (B) Pictures of the biocrust samples. (C) Picture of a bulk soil sample. (D) Full view of Location 1. The diversity of biocrusts can be observed. GPS coordinates of the samples are available upon reasonable request, since the natural resources of the Tabernas Desert are protected by regional rules.
