# Supplementary material for: A Round Trip to the Desert: In situ Nanopore Sequencing Informs Targeted Bioprospecting
Source: Front Microbiol. 2021 Dec 13;12:768240. doi: 10.3389/fmicb.2021.768240 (PMC8710813; doi:10.3389/fmicb.2021.768240)
Supplement: Supplementary file 1 [file Data_Sheet_1.zip › Supplementary Table S7.PDF]

Supplementary Table 7. Mobile laboratory setup. List of main reagents, fungible and equipment used.

Instrument	Description	Units
Laptop	MSI GF63 Thin 9SC-047XES laptop (CPU: Intel Corei7-9750H, 6 core, 12 threads; RAM: 16GB; SSD: 512 Gb; Graphics Card: GeForce GTX 1650)	2
MinION	MinION Mk1B (ONT, Oxford, UK)	1
Flow cell	R9.4.1 MinION flow cell (ONT, Oxford, UK, Cat. No.: FLO-MIN106D)	2
Thermoblock	24 tubes (1.5 mL) thermoblock Labnet 596111 (Labnet, Madrid, Spain)	1
Horizontal vortex	Horizontal vortex for 24 tubes (Selecta J.P., Barcelona, Spain)	1
Microcentrifuge	12 tubes (1.5 mL); 13400 rpm max.; Minispin eppendorf F45-12-11 (Eppendorf, Hamburg, Germany)	1
Qubit	Qubit™ 2.0 Flex Fluorometer (Thermo Fisher, Waltham, United States, Cat. No.: Q33327)	1
Thermocycler	Mastercycler Eppendorf 5332 (Eppendorf, Hamburg, Germany)	1
Pipettes	10, 100 and 1000 μL pipettes	1
Bunsen burner	-	1
**Fungible & reagents**	**Description**	**Units**
Qubit DNA Kit	x1 dsDNA High-Sensitivity Assay kit (Thermo Fisher, Waltham, United States, Cat. No.: Q33230)	1
Pipette tips	10, 100 and 1000 μL x96 pipette tips	2
DNA extraction kit	DNEasy Power Soil Kit (QIAGEN, Germany, Cat. No.: 12888)	1
16S primers	S-D-Bact-0008-a-S-16 and S-D-Bact-1492-a-A-16. Modified with ONT Universal tags.	-
PCR mix	NZYTaq II 2x Green Master Mix (NZYTech, Lisboa, Portugal, Cat. No.: MB358)	1
Purification kit	NucleoMag kit for PCR clean up with magnetic beads (Macherey-Nagel, Germany, Cat. No.: 744100.4).	1
ONT barcoding kit	PCR Barcoding Expansion Pack 1-96 (ONT, Oxford, UK, Cat. No.: EXP-PBC096)	1
ONT ligation kit	Ligation Sequencing Kit (ONT, Oxford, UK, Cat. No.: SQK-LSK109)	1
End-prep kit	NEBNext FFPE DNA Repair Mix (New England Biolabs, Ipswich, US, Cat. No.: M6630)	1
Flow cell wash kit	Flow Cell Wash Kit (ONT, Oxford, UK, Cat. No.: EXP-WSH004)	1
