# Supplementary material for: A Round Trip to the Desert: In situ Nanopore Sequencing Informs Targeted Bioprospecting
Source: Front Microbiol. 2021 Dec 13;12:768240. doi: 10.3389/fmicb.2021.768240 (PMC8710813; doi:10.3389/fmicb.2021.768240)
Supplement: Supplementary file 1 [file Data_Sheet_1.zip › Supplementary File 1.ZIP!/Supplementary-File1.html]

Supplementary File 1: Raw Spaghetti output. Spaghetti: In situ analysis of 16S rRNA nanopore sequencing data


# Supplementary File 1: Raw Spaghetti output.

#### Spaghetti: In situ analysis of 16S rRNA nanopore sequencing data

#### Note that some figures may not be visualized correctly on your browser

#### November, 2020

# Library Size Summary

Library size by sample:

```
##   X1.1   X1.2   X1.3   X1.4   X1.7   X1.8   X2.1   X2.2   X3.1   X3.2   X4.1 
## 124854  91548 118181 122073 108614  92391  83343  50051  98748 102995 118116 
##   X6.2    c.1    c.3 
##  85766 102640 128282
```

Summarized library size:

```
##    Min. 1st Qu.  Median    Mean 3rd Qu.    Max. 
##   50051   91759  102818  101972  118165  128282
```

---


---

# alpha-diversity

Species level (not normalized):

Species level (rarefied to the lower sample):

Genus level (not normalized):

Genus level (rarefied to the lower sample):

---

# beta-diversity

PCoA - Bray Curtis - Species level:

PCoA - Bray Curtis - Genus level:

---

# Top Phyla

# Top Genera

# UV-resistant bacteria:

UV-resistant and/or pigment-producing bacteria have been extensively described in the literature. So, we can know beforehand which taxa to search in this dataset. As the resolution of ONT 16S rRNA sequencing do not reach the species level (even using the entire 16S), we will search at the genus and family level.

## Taxa of interest:

- **Genus level**:
  - Tanner (2020): *Hymenobacter*, *Deinococcus*, *Arthrobacter*, *Alcaligenes*, *Sphingomonas*, *Curtobacterium*, *Microbacterium*, *Kineococcus*, *Methylobacterium*, *Rubrobacter*, *Modestobacter*, *Rubellimicrobium*, *Planomicrobium*, *Bacillus*, *Rhodobacter*, *Chryseobacterium*
  - Deng *et al.* (2016): *Micrococcus*
  - Paulino-Lima *et al.* (2016): *Kocuria*, *Pontibacter*, *Geodermatophilus*, *Cellulomonas*
  - Yu *et al.* (2013): *Knoellia*, *Lysobacter*, *Nocardioides*, *Paracoccus*, *Pontibacter*, *Rufibacter* and *Microvirga*
  - Etemadifar *et al.* (2015): *Exiguobacterium*
- **Family level** (taxonomy as in Silva 138)
  - *Hymenobacteraceae*: *Hymenobacter*, *Pontibacter*, *Rufibacter*
  - *Deinococcaceae*: *Deinococcus*
  - *Micrococcaceae*: *Arthrobacter*, *Micrococcus*, *Kocuria*
  - *Microbacteriaceae*: *Curtobacterium*, *Microbacterium*
  - *Alcaligenaceae*: *Alcaligenes*
  - *Sphingomonadaceae*: *Sphingomonas*
  - *Kineosporiaceae*: *Kineococcus*
  - *Beijerinckiaceae*: *Methylobacterium-Methylorubrum*, *Microvirga*
  - *Rubrobacteraceae*: *Rubrobacter*
  - *Geodermatophilaceae*: *Modestobacter*, *Geodermatophilus*
  - *Rhodobacteraceae*: *Rubellimicrobium*, *Rhodobacter*, *Paracoccus*
  - *Planococcaceae*: *Planomicrobium*
  - *Bacillaceae*: *Bacillus*
  - *Weeksellaceae*: *Chryseobacterium*
  - *Cellulomonadaceae*: *Cellulomonas*
  - *Intrasporangiaceae*: *Knoellia*
  - *Xanthomonadaceae*: *Lysobacter*
  - *Nocardioidaceae*: *Nocardioides*
  - *Exiguobacteraceae*: *Exiguobacterium*
  - *Trueperaceae*: *Truepera*

Note: *Rufibacter* and *Microvirga* are NOT in GreenGenes 13\_8, but they are included in Silva 138.

(We finally use Silva 138).

---

## Percentage of UV-resistant bacteria (Genus Level)

**Not grouped:**

**Grouped:**

**Richness of UV-bacteria (Presence/Absence):**

*samples has been sorted by total richness (presence of UV-resisstant bacteria)*

**Abundance + richness of UV-bacteria (all together):**

*Note: Samples are sorted by the total number of UV-resistant bacteria found (UV Bacteria)* *Note 2: Rarefaction to the lower library size has been performed in order to calculate richness*

---

**OTU-like tables have been written for Species, Genera & Phylum Level.**
